# Meeting of the Alumni of the Albany Medical College

**Published:** 1874-12

**Authors:** 


### Meeting of the Alumni of the Albany Medical College.

The Second Annual Meeting of the "Association of the Alumni of the Albany Medical College," was held in the city of Albany, on Tuesday Dec., 22d. The following officers were elected for the ensuing year:

*President.*—Dr. Jno. H. Beech, (41) Michigan.

*Vice Presidents.*—Drs. A. D. Hull, (41) N. Y., B. N. Mynders, (53) N. Y.,

Alex. Shiland, (53) N. Y., Sol. Van Etten, (53) N. Y., Chas. L. Spencer, (57) Mass.

*Secretary.*—D. Willis G. Tucker, (70) N. Y.

*Treasurer.*—G. L. Ullman, (71) N. Y.

*Executive Committee.*—Drs. H. D. Didama, (46); Wm. S. Young, (41); Jas. H. Scoon, (49); Jas. S. Bailey, (53); M. H. Burton (53); Jno. H. Hill, (53); Charles. H. Burbeck, (59); N. P. Ten Eyck, (66); J. H. Blatner, (72); Oscar Myers, (73).

The Address of the retiring President, Prof. Didama, of the Syracuse University, was listened to with much interest. In the evening the Association partook of its Annual Supper at the Delevan House, about one hundred and fifty graduates being present.

